# Damage evolution law of multi-hole blasting igneous rock and quantitative evaluation model of damage degree based on fractal theory and clustering algorithm

**DOI:** 10.1038/s41598-024-66126-6

**Published:** 2024-07-10

**Authors:** Hongbin Zhao, Shihao Tu, Kaijun Miao, Long Tang, Jieyang Ma, Benhuan Guo

**Affiliations:** School of Mines, China University of Mining and Technology, Xuzhou, 221116 China

**Keywords:** Igneous rock, Rock damage evolution, Blasting effect evaluation, Box-counting dimension, K-means clustering, Civil engineering, Petrology, Structural geology, Applied mathematics

## Abstract

The geological phenomenon of igneous rock invading coal seam is widely distributed, which induces mining risk and affects efficient mining. The pre-splitting blasting method of igneous rock is feasible but difficult to implement accurately, resulting in unnecessary safety and environmental pollution risks. In this paper, the blasting model with penetrating structural plane and the multi-hole blasting model with different hole spacing were established based on the Riedel–Hiermaier–Thoma (RHT) damage constitutive to explore the stress wave propagation law under detonation. The damage cloud diagram and damage degree algorithm were used to quantitatively describe the spatio-temporal evolution of blasting damage. The results show that the explosion stress wave presents a significant reflection stretching effect under the action of the structural plane, which can effectively aggravate the presplitting blasting degree of the rock mass inside the structural plane. The damage range of rock mass is synchronously evolved with the change of blasting hole spacing. The blasting in the igneous rock intrusion area of the 21,914 working face is taken as an application example, and the damage degree of rock mass is reasonably evaluated by the box-counting dimension and K-means clustering method, which proves the effectiveness of the blasting scheme and provides reference value for the implementation of related blasting projects.

## Introduction

The intrusion of igneous rock into coal-bearing strata is a common geological phenomenon. The intrusive rock layer has important effect on the thickness of coal seam, the degree of coal metamorphism and the implementation of safe mining technology, which is specifically manifested in the change of the physical and mechanical properties, structure and components of coal seam^1–3^. When facing the intrusion of large area igneous rock, it will hinder the normal coal cutting work of the coal cutter, so it is necessary to take reasonable technical means to carry out advanced cracking or crushing treatment.

As a common method of rock mass fragmentation, blasting is widely used in many engineering projects, but it is difficult to form a unified blasting scheme due to the complexity of rock mass itself and the different forms of explosion energy transfer. In view of the pre-splitting blasting engineering practice in igneous rock intrusion area, the layout of blasting hole positions will be significantly affected by factors such as intrusion range and weak interlayer characteristics at the junction of coal and rock. Therefore, it is necessary to focus on the influence of structural plane on pre-splitting blasting effect and the damage evolution law of multi-hole blasting rock mass, domestic and foreign scholars have done a lot of research work on these two problems at present. Lou et al.^4^ studied the crack propagation law and stress wave propagation law at different positions of joints under explosive load, and defined the transmission-reflection energy ratio of the joint to measure the fracture effect of the inner and outer rock masses. Raina et al.^5^ introduced the blasting damage index to quantitatively describe the response mechanism of blasting damage degree of rock mass to structural plane spacing, dip angle, blasting hole depth, spacing, charge concentration and other factors. Li et al.^6^ adopted CDEM method to explore the influence of joint strength, stiffness and spacing on stress wave propagation and blasting effect. Miranda et al.^7^ analyzed the effect of rough or smooth joint characteristics on acoustic wave propagation through acoustic wave test experiments. Li et al.^8^ explored the propagation law of stress wave at single and two filling joints by improving SHPB and found the thickness of filling joints and material properties would affect the transmission and reflection ratio of stress wave.

For the damage evolution law of multi-hole blasting rock mass, model experiments and similar simulation methods are used to carry out scientific research. Cao et al.^9^ discussed the effects of hole spacing and extra freedom on blasting damage during double-hole blasting, and described the spatiotemporal evolution of rock damage around the hole with the change of effective damage rate. Jayasinghe et al.^10^ proposed a three-dimensional coupled smooth particle hydrodynamics and finite element methods to study the influence of discontinuous high in-situ stress on the evolution of rock multi-hole blasting damage. Chen et al.^11^ carried out fifteen multi-hole blasting tests on rectangular concrete blocks under different constraints by orthogonal test method, then explored the fracture characteristics of multi-hole blasting rock under the coupling action of static stress and delay time. Based on the engineering background of gas extraction in low permeability coal seam, Liu et al.^12^ developed single-hole, double-hole and pilot-hole blasting tests to discuss the influence of hole arrangement on static blasting. Shen et al.^13^ prepared rock samples with different inclined angles of cracks, and adopted a new digital laser dynamic caustics test system to explore the dynamic propagation law of cracks in models with inclined defects. In order to analyze the blasting damage effect, it is necessary to quantitatively characterize the damage degree. Qiao et al.^14^ proposed a damage area evaluation method based on image processing technology to accurately calculate the blasting damage degree. Deep learning algorithms were used to predict the fracture expansion path and the trajectory characteristics of the broken block, and then evaluate the blasting effect of the rock mass^15^. In addition, digital core and CT 3D reconstruction technology have also begun to be applied in the blasting test to quantitatively characterize the blasting damage effect by extracting the pore fracture structure in the rock mass^16,17^.

It can be seen from the above research status that the existing research mainly focuses on the theoretical research of explosion stress wave propagation and damage failure mode. Most researches have not carried out specific analysis in combination with engineering practice. The numerical model under different working conditions was established by LS-DYNA and the model material parameters were calibration. The stress wave propagation and damage evolution law of rock mass with structural plane and multi-hole blasting were studied. The damage cloud diagram of the model was used to describe the rock blasting process, meanwhile the change of damage value was adopted to quantitatively characterize the temporal and spatial evolution process of the rock mass. The principle of blasting hole layout in igneous rock intrusion area was summarized to design the blasting scheme of 21,914 working face (igneous rock intrusion area). The results showed that the effect of blasting fracturing rock mass is good, which can provide reference value for the implementation of related blasting projects.

## Calculation model establishment and parameter setting

In order to ensure the safe mining of the working face in the igneous rock intrusion section, the large area of igneous rock needs to be crushed by pre-crack blasting method. Due to the different occurrence of the structural plane of the rock mass and the explosion energy transfer form, the blasting process is difficult to accurately control, while the blasting effect is uneven. Therefore, ANSYS/LS-DYNA numerical simulation software is used to establish the through structural plane blasting model and the multi-hole blasting model. The results reveal the transmission law of explosion stress wave and the failure characteristics, which is valuable for blasting hole arrangement and blasting parameter optimization design in igneous rock intrusion area.

### Calculation model establishment

#### Blasting model of rock mass with penetrating structural plane (see Fig. 1)

**Figure 1:**
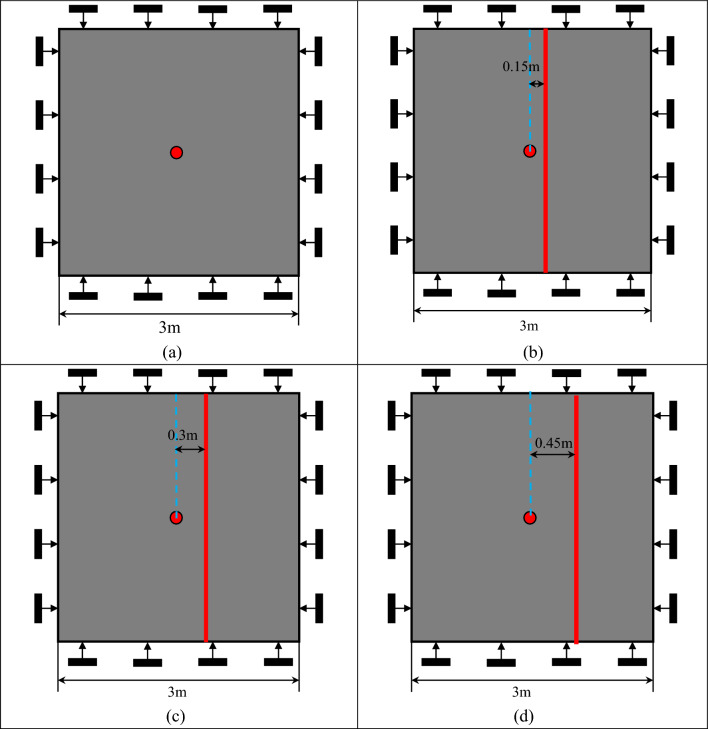
Complete rock model and rock model with structural plane. (**a**) Without structural plane, (**b**) 0.15 m from the blast hole, (**c**) 0.3 m from the blast hole, (**d**) 0.45 m from the blast hole.

The calculation models of intact rock mass and rock mass with structural plane are established respectively to analyze the law of explosion stress wave propagation, rock mass damage and crack extension. The size of the model is 3.0 × 3.0 m, the blasting hole is located in the center, and the radius d = 0.02 m. The plane strain model is used for numerical study in this paper, which implies the assumption of infinite velocity of detonation along the depth of borehole and infinite charge length. The model is constrained by displacement, and non-reflective boundary conditions are applied all around to eliminate the influence of artificial boundary reflection waves. Large stiffness values and cohesion forces are applied to the structural surface to ensure that no internal deformation and damage occurs. The structural plane is set according to different simulation schemes, and the four boundaries of the model are set without reflection boundaries to simulate the infinite range of rocks. The distance between the structural plane and the blasting hole is 0.15 m, 0.3 m and 0.45 m, and the width of the structural plane is 0.01 m, as shown in the figure. The model consists of four parts: explosive, air, rock mass and igneous structural plane. Among them, explosives and air are common nodes, while rocks and igneous structural planes are common nodes.

#### Multi-hole blasting model of rock mass (see Fig. 2)

**Figure 2:**
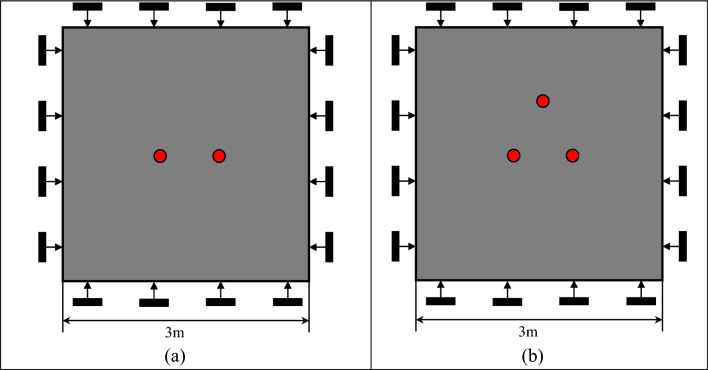
Multi-hole blasting model of rock mass. (**a**) Double-hole blasting, (**b**) triple-hole blasting.

Double-hole and multi-hole models with different blasting hole spacing are established at first. According to the theory of blasting crushing zone, crack zone expansion, the blasting hole spacing is designed as 0.2 m, 0.4 m, 0.6 m, 0.8 m and 1.0 m, and the other settings are the same as model (1).

### Model material parameter setting

#### Explosive

The MAT_HIGH_EXPLOSIVE_BURN model and JWL state equation are used to simulate explosive explosion in LS-DYNA^18^. The main parameters include explosive density *ρ*, detonation velocity *D*, explosive detonation parameters *A, B, R*_1_*, R*_2_*, ω*, initial specific internal energy *E*_0_ and detonation pressure *P* (as shown in Table 1). The expression is given in Eq. (1).

1 $$P = \left( {1 - \frac{\omega }{{R_{1} V}}} \right)e^{{ - R_{1} V}} + B\left( {1 - \frac{\omega }{{R_{2} V}}} \right)e^{{ - R_{2} V}} + \frac{{\omega E_{0} }}{V}$$

**Table 1:** Physical parameters of explosive model.

Parameter	*ρ* (g/cm^3^)	*D* (m/s)	*A* (GPa)	*B* (GPa)	*R*_1_	*R*_2_	*ω*	*E*_0_ (GPa)
Value	1.32	6690	586	21.6	5.81	1.77	0.282	7.38

#### Air

The air is described by the MAT_NULL model and EOS_LINEAR_POLYNOMIAL equation of state. The linear polynomial equation of state is *P* = *C*_0_ + *C*_1_*μ* + *C*_2_*μ*^2^ + *C*_3_*μ*^3^ + (*C*_4_ + *C*_5_*μ* + *C*_6_*μ*_2_)*E*_0_. *μ* is the specific volume, and *C*_0_*-C*_6_ is the air material parameters, values refer to Jayasinghe’s literature^10^, as seen in Table 2.

**Table 2:** Physical parameters of air model.

Parameter	*ρ* (g/cm^3^)	*C*_0_	*C*_1_	*C*_2_	*C*_3_	*C*_4_	*C*_5_	*C*_6_	*E*_0_ (Pa)
Value	1.293	0	0	0	0	0.4	0.4	0	2.5 × 10^5^

#### Structural plane

The influence of the structural plane on the propagation of the blasting crack is related to the properties of the rock mass on both sides of the structural plane, the filling medium, and the thickness^19–21^. This paper mainly discusses the influence of the relative position of the structural plane from the blasting hole to the blasting effect. Therefore, the thickness of the structural plane is unchanged, and the filling medium is often weak material^22^. The PK constitutive model of weak rock stratum is adopted, and its parameter values are shown in Table 3.

**Table 3:** Physical parameters of structural plane model.

Parameter	*ρ* (g/cm^3^)	*E*_0_ (GPa)	*μ*	*σ* (GPa)	*E*_*tan*_	β
Value	1.6	20	0.3	0.025	0.04	0.5

#### Rock mass

Considering the influence of the confining pressure, strain rate, strain hardening, damage softening and other factors on the failure strength of rock materials under the action of blasting dynamic load, the RHT constitutive model is selected for rock mass materials. The RHT model contains 34 model parameters need to be imported, some of which can be obtained by experimental measurement or theoretical calculation. The remaining parameters can be obtained by the original literature of the RHT model, as shown in Table 4.

**Table 4:** Physical parameters of rock mass model (RHT).

Parameter	Value	Parameter	Value
Mass density *ρ*_0_ (g/cm^3^)	2.62	Elastic shear modulus *G* (GPa)	21.19
Compressive strength *f*_*c*_ (MPa)	158.36	Compressive strain rate exponent *βc*	0.032
Initial porosity *α*_0_	1.0	Tensile strain rate exponent *β*_*t*_	0.036
Polynomial coefficient *A*_1_ (GPa)	35.27	Reference compressive strain rate ε̇_0_^*c*^ (s^−1^)	3.0 × 10^−5^
Polynomial coefficient *A*_2_ (GPa)	39.58	Reference tensile strain rate ε̇_0_^*t*^ (s^−1^)	3.0 × 10^−6^
Polynomial coefficient *A*_3_ (GPa)	9.04	Break compressive strain rate ε̇^*c*^ (s^−1^)	3.0 × 10^25^
Parameter for polynomial EOS *B*_0_	1.22	Break tensile strain rate ε̇^*t*^ (s^−1^)	3.0 × 10^25^
Parameter for polynomial EOS *B*_1_	1.22	Damage parameter *D*_2_	1
Parameter for polynomial EOS *T*_1_ (GPa)	25.7	Porosity exponent *N*_*p*_	3
Parameter for polynomial EOS *T*_2_ (GPa)	0	Failure surface Parameter *N*	0.76
Compaction pressure *P*_*comp*_ (GPa)	6.0	Relative tensile strength *f*_t_*	0.1
Relative shear strength *f*_s_*	0.18	Failure surface Parameter *A*	2.44
Lode angle dependence factor *Q*_0_	0.68	Lode angle dependence factor *B*	0.0105
Damage parameter *D*_1_	0.04	Shear modulus reduction factor *ξ*	0.50
Lode angle dependence factor *ε*_*p*_^*m*^	0.01	Residual surface parameter *A*_*f*_	1.62
Residual surface parameter *N*_*f*_	0.61	Crush pressure *P*_*el*_ (MPa)	105.5
Compressive yield surface parameter *g*_c_*	0.53	Tensile yield surface parameter *g*_t_*	0.70

**Figure 3:**
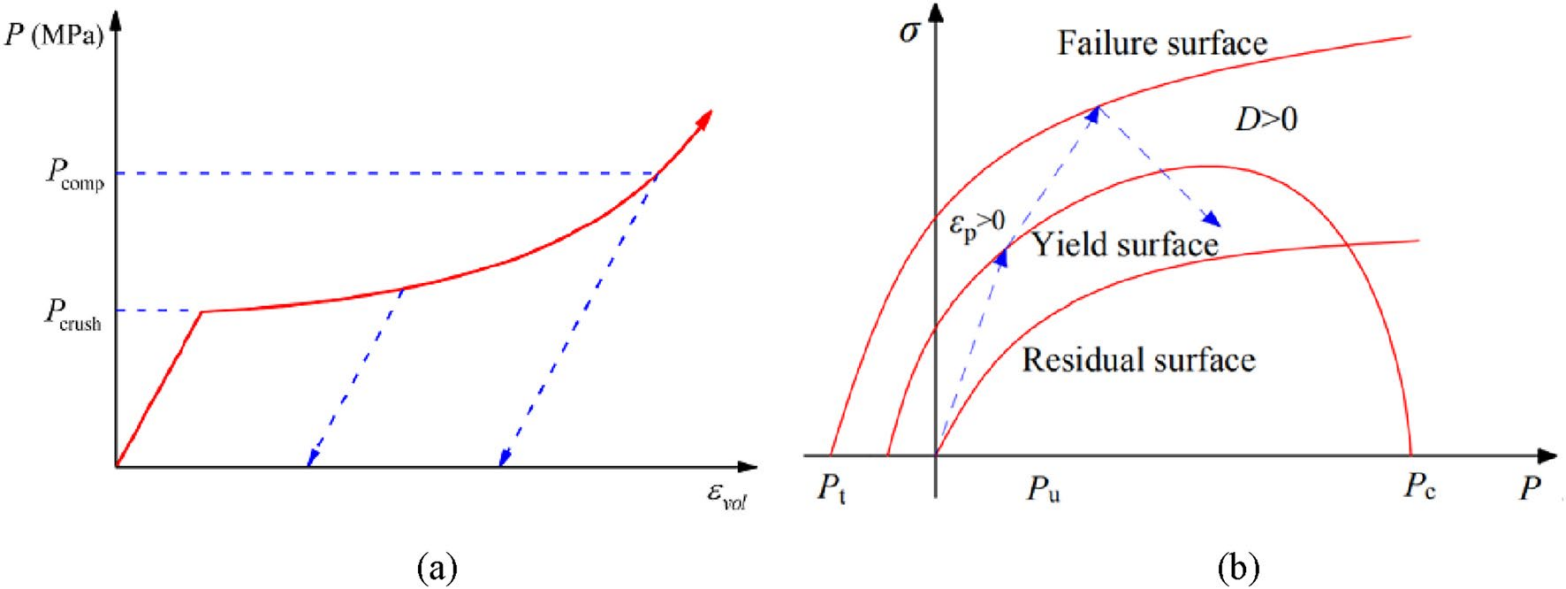
Introduction to the RHT constitutive model^35^. (**a**) Schematical description of the p-α equation of state. (**b**) Stress limit surfaces and loading scenario.

The RHT model considers the effect of the third invariant of stress tensor on the shape of damage surface, which can qualitatively determine the strain type and stress state of the material^22^. In the compaction model, the model is elastic when the pressure value is lower than the pore collapse pressure. Once the pressure exceeds the pore crushing pressure, the pore collapse reduces the bulk stiffness of the material, and the pressure has a nonlinear relationship with the bulk strain, as seen in Fig. 3a. In addition, the strength model is described by three limit surfaces, namely yield surface, failure surface and residual strength surface, as shown in Fig. 3b. The arrow in Fig. 3b depicts the typical loading process of the material, which is divided into three stages: elastic deformation stage, plastic hardening stage and softening damage stage.

Firstly, the material density *ρ*_0_, uniaxial compressive strength *f*_*c*_, shear modulus *G* and initial porosity *α*_0_ are obtained by rock physical and mechanical tests. The relative tensile strength *f*_t_^*^ and relative shear strength *f*_s_^*^ are determined by calculating the tensile strength and shear strength to compressive strength^23,24^.

Secondly, the strain rate has a significant effect on the strength of rock^25^. The relationship between the strain rate correlation coefficient and the strength given in the RHT model is shown in Eq. (2)^26^. In addition, the key parameters of the state equation of rock mass compaction in RHT model can be obtained from the existing literature^27–29^.

2 $$F_{r} \left( {\dot{\varepsilon }_{p} ,P} \right) = \left\{ {\begin{array}{*{20}l} {\left( {\frac{{\dot{\varepsilon }_{p} }}{{\dot{\varepsilon }_{0}^{c} }}} \right)^{{\beta_{c} }} } \hfill & {P \ge {{f_{c} } \mathord{\left/ {\vphantom {{f_{c} } 3}} \right. \kern-0pt} 3}} \hfill \\ {\frac{{P + {{f_{t} } \mathord{\left/ {\vphantom {{f_{t} } 3}} \right. \kern-0pt} 3}}}{{{{f_{c} } \mathord{\left/ {\vphantom {{f_{c} } {3{{ + f_{t} } \mathord{\left/ {\vphantom {{ + f_{t} } 3}} \right. \kern-0pt} 3}}}} \right. \kern-0pt} {3{{ + f_{t} } \mathord{\left/ {\vphantom {{ + f_{t} } 3}} \right. \kern-0pt} 3}}}}}\left( {\frac{{\dot{\varepsilon }_{p} }}{{\dot{\varepsilon }_{0}^{c} }}} \right)^{{\beta_{c} }} - \frac{{P - {{f_{c} } \mathord{\left/ {\vphantom {{f_{c} } 3}} \right. \kern-0pt} 3}}}{{{{f_{c} } \mathord{\left/ {\vphantom {{f_{c} } {3{{ + f_{t} } \mathord{\left/ {\vphantom {{ + f_{t} } 3}} \right. \kern-0pt} 3}}}} \right. \kern-0pt} {3{{ + f_{t} } \mathord{\left/ {\vphantom {{ + f_{t} } 3}} \right. \kern-0pt} 3}}}}}\left( {\frac{{\dot{\varepsilon }_{p} }}{{\dot{\varepsilon }_{0}^{t} }}} \right)^{{\beta_{t} }} } \hfill & {{{ - f_{t} } \mathord{\left/ {\vphantom {{ - f_{t} } 3}} \right. \kern-0pt} 3} < P < {{f_{c} } \mathord{\left/ {\vphantom {{f_{c} } 3}} \right. \kern-0pt} 3}} \hfill \\ {\left( {\frac{{\dot{\varepsilon }_{p} }}{{\dot{\varepsilon }_{0}^{t} }}} \right)^{{\beta_{t} }} } \hfill & {P \le {{ - f_{t} } \mathord{\left/ {\vphantom {{ - f_{t} } 3}} \right. \kern-0pt} 3}} \hfill \\ \end{array} } \right.$$

where $\dot{\varepsilon }_{0}^{c}$ is the reference strain rate under compression; $\dot{\varepsilon }_{0}^{t}$ is the reference strain rate under tension; *P* is the pressure; *β*_*c*_ and *β*_*t*_ are the material constants for compression and tension, *β*_*c*_ = 4/(20 + 3*f*_*c*_), *β*_*t*_ = 2/(20 + *f*_*c*_).

Thirdly, the failure surface parameters *A* and *N*, crush pressure *p*_*el*_, lode angle dependence factors *Q*_0_ and *B*, minimum damaged residual strain *ε*_*p*_^*m*^ and damage parameter *D*_1_ and *D*_2_ must be determined, all the parameters are obtained from Yu, Xie and Wang experiment analysis results^30–33^. The remaining parameters can be directly used from the RHT raw model data, including tensile yield surface parameter gt*, compressive yield surface parameter gc*, etc^34,35^.

### Calibration of the RHT model

Banadaki used model test method to study the cracks generated by explosion stress wave in rock, and obtained good test results. Among them, the granite sample selected by Banadaki belongs to igneous rock. Compared with the rock mass in the igneous intrusion area of the research object in this paper, the basic physical and mechanical parameters of the two are similar. Therefore, this paper intends to use the numerical simulation method to repeat the test, and compare the Banadaki model test results with the numerical simulation results to verify the feasibility of the numerical simulation method and the applicability of the model parameters.

**Figure 4:**
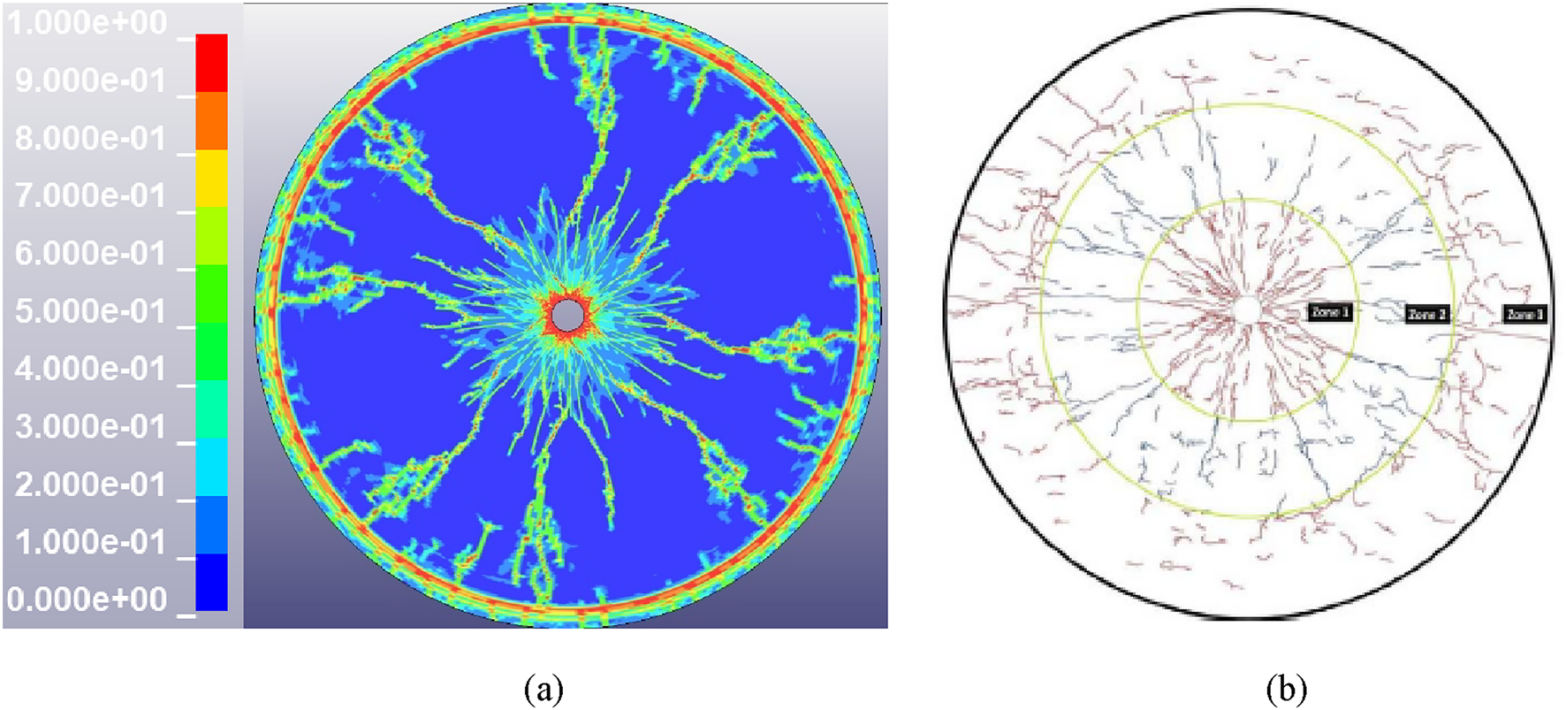
Final failure pattern under explosion stress wave. (**a**) Simulation result. (**b**) Test result^26^.

After the detonation of the explosive, a large amount of plastic deformation is produced along the blasting hole, and a compression fracture zone is generated around the blasting hole. Then the energy generated by the explosion continues to be transmitted in the form of stress wave in the rock. Due to the low tensile strength of the rock, the tensile component of the stress wave causes tensile damage to the rock. The reflected wave is generated while the stress wave is transmitted to the free surface. Under the superposition of the incident wave and reflected wave, the rock near the free surface will peel off and penetrate the radial development crack. Finally, the rock damage crack extends from the blasting hole to the free surface during the whole explosion process, as shown in the Fig. 4a.

In the numerical simulation of rock blasting, the finer and more regular the mesh is, the closer the crack is to the real situation. The mapping method is used to divide the grid for a smaller computational load of numerical simulation. This will make the cracks at the edge of the rock sample in the numerical simulation thicker and denser than those in the experiments, but the crack distribution range and characteristics obtained by the numerical simulation and the test are similar in general, as seen in the Fig. 4. The above findings show that the numerical simulation results are in good agreement with the experimental results, and the model can accurately simulate the damage and failure characteristics of rock.

## Numerical simulation results and analysis

### Influence of structural plane on blasting effect of rock mass

**Figure 5:**
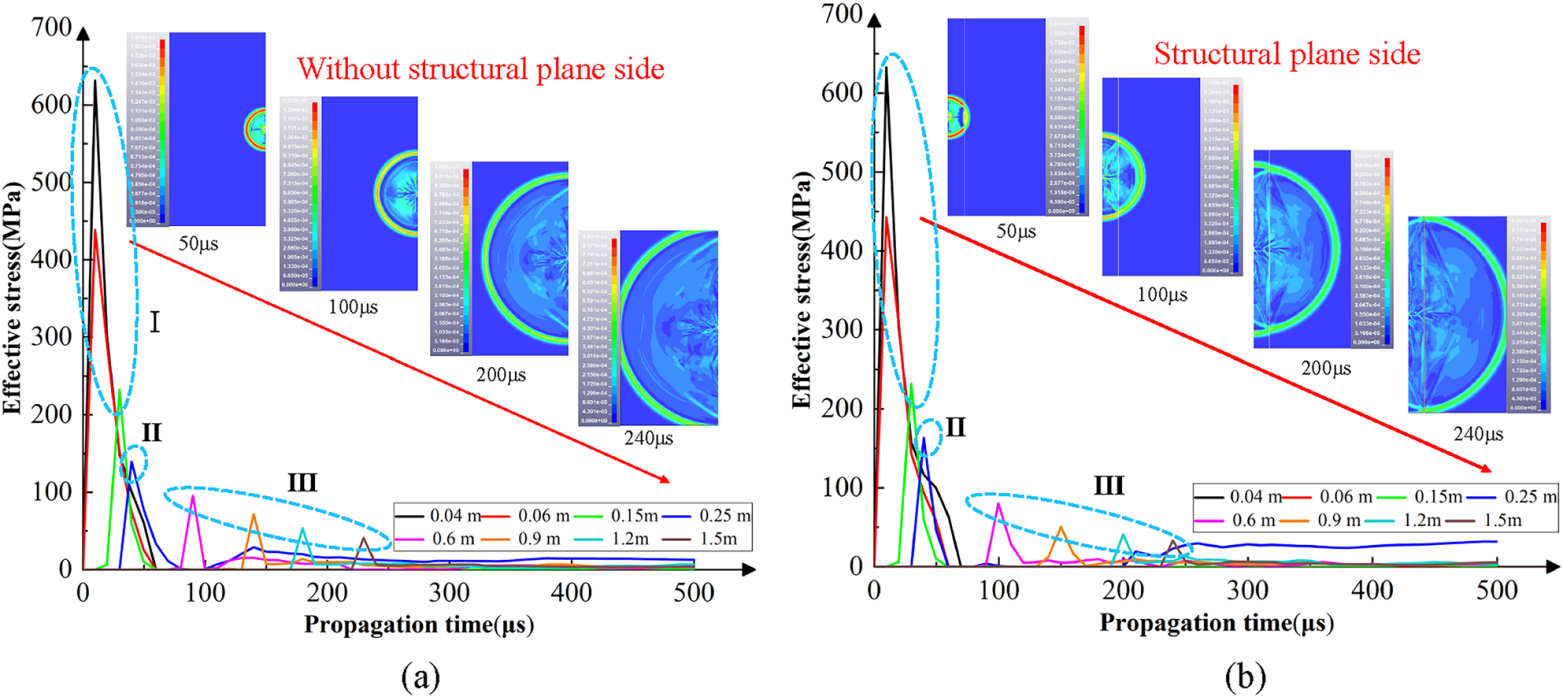
Propagation process of blasting stress wave and stress distribution rule of measurement point in rock mass with structural plane. (**a**) Without structural plane side. (**b**) With structural plane side.

Taking the model with a structural plane of 0.25 m away from the blasting hole as an example, eight measuring points are selected from the center of the blasting hole to the left and right to monitor the characteristics of the explosion stress wave, which are 0.04, 0.06, 0.15, 0.25, 0.6, 0.9, 1.2 and 1.5 m away from the center of the blasting hole, as seen in Fig. 5. The explosion stress wave propagates outward in the form of geometric attenuation. On the structural plane side, the incident stress wave is divided into reflected wave and transmitted wave, and the stress value is discontinuous at the structural plane. According to the variation characteristics of effective stress at each measurement point, it can be divided into three sections. Region I: the inner part of the structural plane where the reflected wave has no effect (the distance is less than 0.15 m). Region II: the inner part of the structural plane where the reflected wave has effect (the distance is 0.15–0.25 m). Region III: the outer part of the structural plane where the transmitted wave has effect (the distance is more than 0.25 m). In region I, the stress wave shows a normal attenuation state and the effective stress variation characteristics on both sides of the blasting hole are consistent. The stress wave propagation law of the structural plane side and the non-structural plane side in region 2 is inconsistent. At the same position from the blasting hole, the effective stress on the side with structural plane is greater than the other. This is because the incident stress waves are divided into reflected and transmitted waves under the action of the structural plane, and this reflection phenomenon will cause stress concentration in the rock mass inside the structural plane. However, the action range of the reflected wave is limited, so the stress variation characteristics of region I and II are different. Region III reflects the propagation law of transmission wave in the rock mass outside the structural plane. It can be found that transmission wave presents a certain delay lag and oscillation attenuation effect, which is manifested as the decrease of the stress peak and the delay of the peak time on the structural plane side at the same position.

On the basis of clarifying the stress wave transmission and reflection effect on the structural plane, the law of crack propagation in the inner and outer rock mass is further analyzed. The damage value from 0 to 1 is used to quantitatively describe the crack propagation and damage degree of the rock mass. The damage value of 0.9–1 is defined as the crushing zone, 0.1–0.9 is defined as the crack zone, which constituted the rock damage zone together, and 0–0.1 is defined as the original rock area (considered as no damage). It can be seen from the figure that the explosion stress wave is reflected at the structural plane, then the rock mass inside the structural plane produces an axial crack which extends vertically and connects with the radial crack generated by the detonation pressure to form an obvious broken or full crack propagation zone.. In addition, part of the stress wave with attenuation energy continues to transmit through the structural plane, resulting in the reduction of the damage degree outside the structural plane.

**Figure 6:**
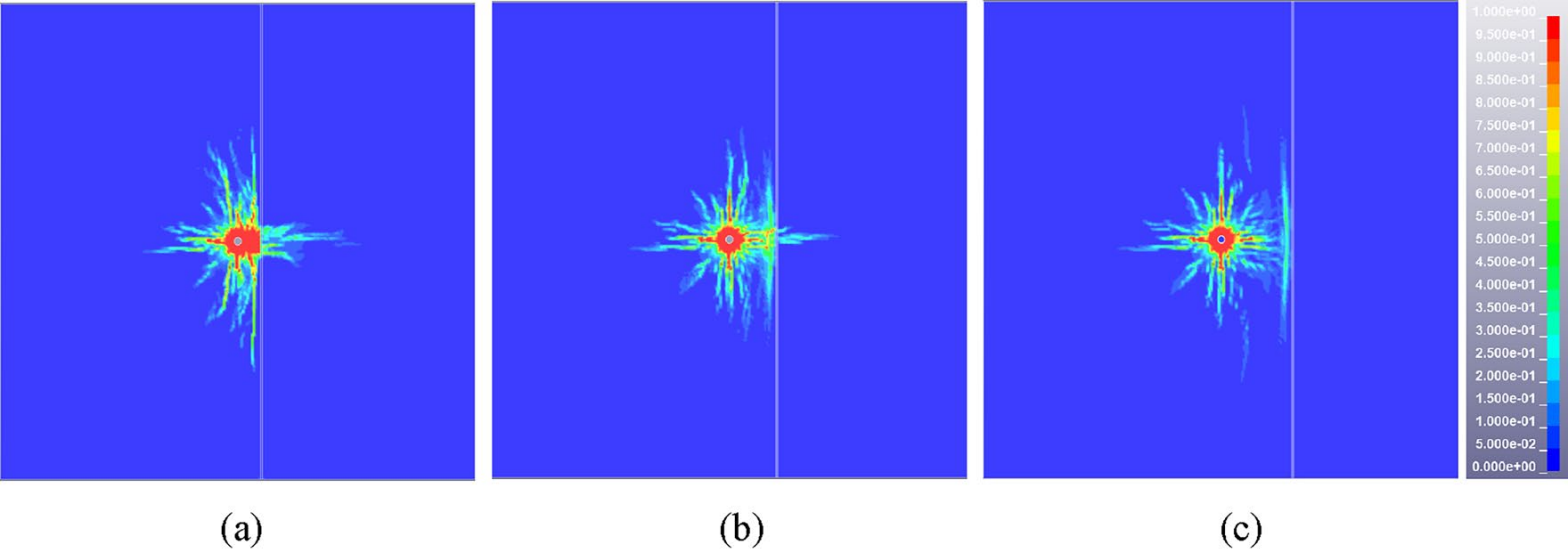
Cloud diagram of rock mass damage distribution under different distance between structural plane and blasting hole. (**a**) 0.15 m, (**b**) 0.3 m, (**c**) 0.45 m.

**Figure 7:**
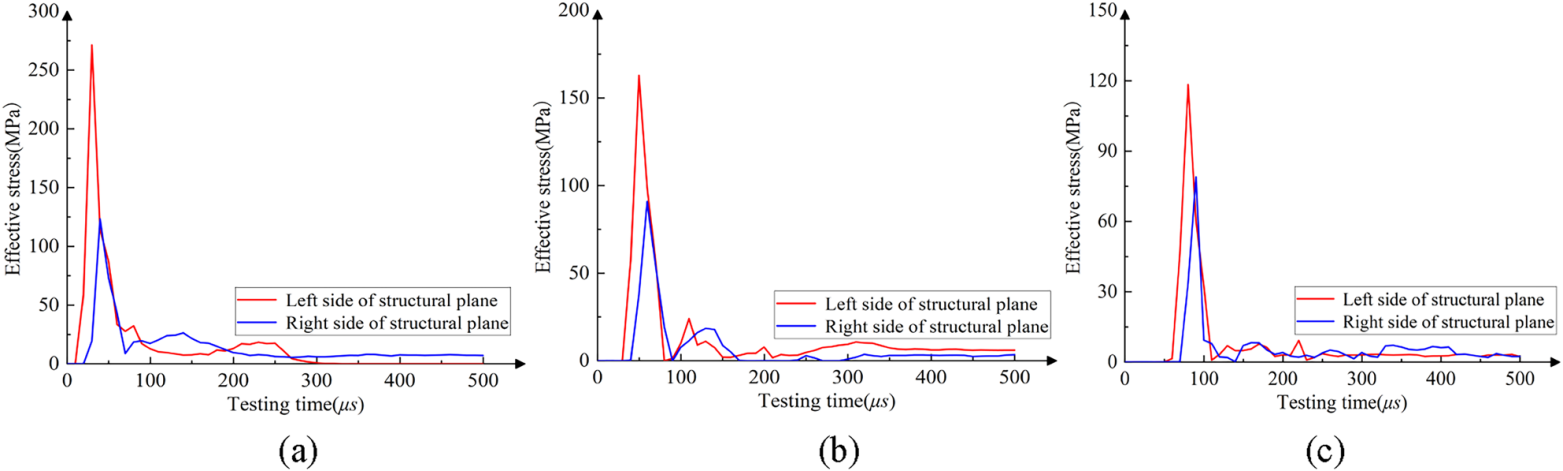
Evolution of effective stress at left and right measuring points of structural plane under different distance between structural plane and blasting hole. (**a**) 0.15 m, (**b**) 0.3 m, (**c**) 0.45 m.

The damage (crack propagation) diagrams of rock mass are extracted from the result files of blasting model with penetrating structural plane, as shown in Fig. 6. When the distance between the blasting hole and structural plane is 0.15 m, the crushing zone is connected with the crack interweaving zone on the inner side of the structural plane, which causes the zone to be completely broken and unable to play a supporting role. When the distance increases to 0.3 m, the crushing zone is not connected with the crack interweaving zone, and the cracks inside the structural plane are fully developed to ensure good rock breaking effect. As the distance extends to 0.45 m, the vertical extension of the layered crack development is not sufficient due to the large attenuation of the explosion stress wave with the propagation distance, resulting in the reduction of the damage area and the poor rock breaking effect. In addition, taking a monitoring point on the left and right sides of the structural plane, the ratio of reflected and transmitted wave to incident wave under different conditions can be obtained, as shown in Fig. 7. The natural dissipation of stress wave is not considered in the calculation process, and the incident wave is equal to the sum of reflected wave and transmitted wave. The ratio of transmitted wave to incident wave is 0.454, 0.558 and 0.667 respectively when the distance is 0.15 m, 0.3 m and 0.45 m, and the ratio of reflected wave to incident wave is 0.546, 0.442 and 0.333, as seen in Table 5. With the increase of the distance between the structural plane and the explosion, the reflection ratio of the stress wave at the structural plane decreases and the transmission ratio increases. In summary, the optimal distance between the blasting hole and the structural plane should be guaranteed to be about 0.3 m when there are structural planes such as joints and contact boundaries. The distance between blasting holes and structural plane can be appropriately increased under the restricted condition of blasting hole numbers, but it should not exceed 0.45 m.

**Table 5:** Ratio of transmitted wave to incident wave at different positions of structural plane.

Distance between structural plane and blasting hole/m	Ratio of transmitted wave to incident wave
0.15	0.454
0.30	0.558
0.45	0.667

### Analysis of damage evolution law and blasting hole location arrangement of multi-hole blasting rock mass

The layout requirements of blasting holes at the structural plane can be clarified through the above analysis. It is necessary to further discuss the damage evolution law of multi-hole blasting rock mass and the arrangement of blasting holes. The spacing of blasting holes is the key factor to determine the blasting effect of igneous rock under the conditions of known rock mechanical properties, explosive parameters, blasting range.

**Figure 8:**
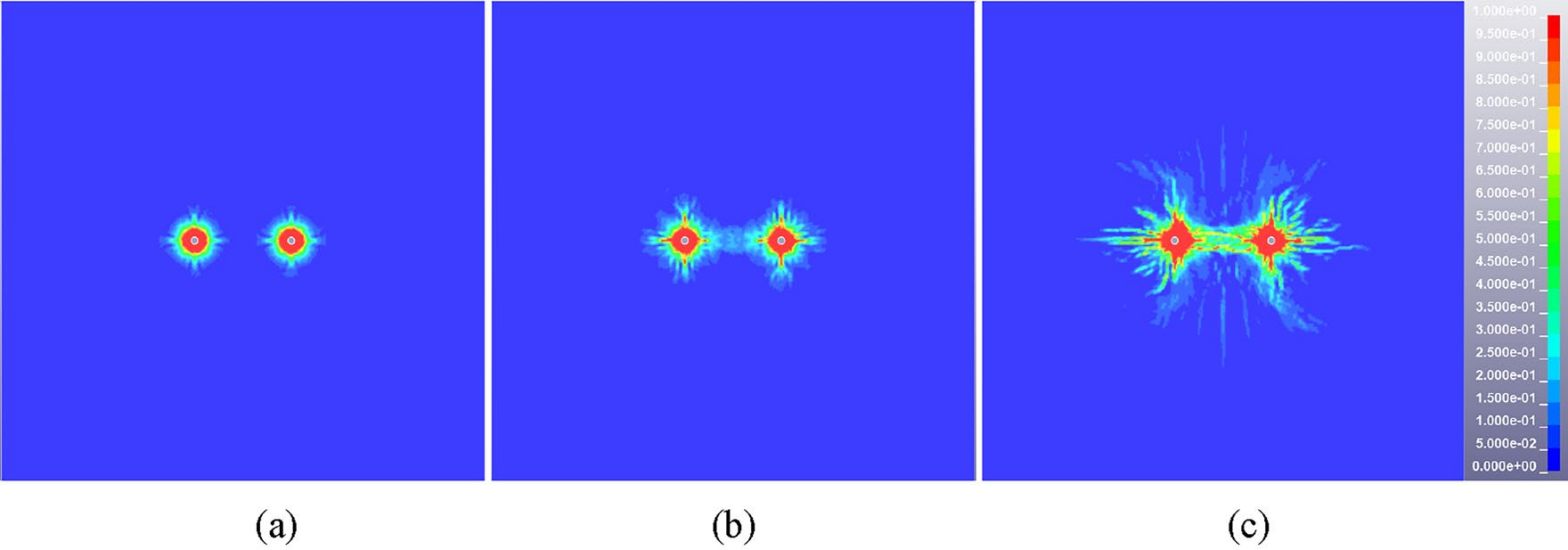
Cloud diagram of damage evolution at typical time for double-hole blasting model (hole spacing 0.6 m). (**a**) 50 μs, (**b**) 100 μs, (**c**) 500 μs.

The damage (crack propagation) diagrams of rock mass are extracted from the result files of multi-hole blasting model. The blasting hole spacing of 0.6 m is taken as an example to illustrate the damage evolution process of double-hole blasting, as shown in Fig. 8. After the initiation of the explosive, the damage range of the rock mass centered on the blast hole expands in all directions. The adjacent gun holes do not affect each other temporarily and the damage range of the rock mass expands separately. The damage area between the two holes starts to connect at 100 μs, while the cracks tend to be parallel to the direction of the connection. Due to the guiding effect of the hole and the stress superposition effect, the damage is preferentially developed between the two holes and the damage degree is greater than other areas. The damage range of rock does not change significantly after 500 μs and presents the characteristics of non-uniform distribution finally.

**Figure 9:**
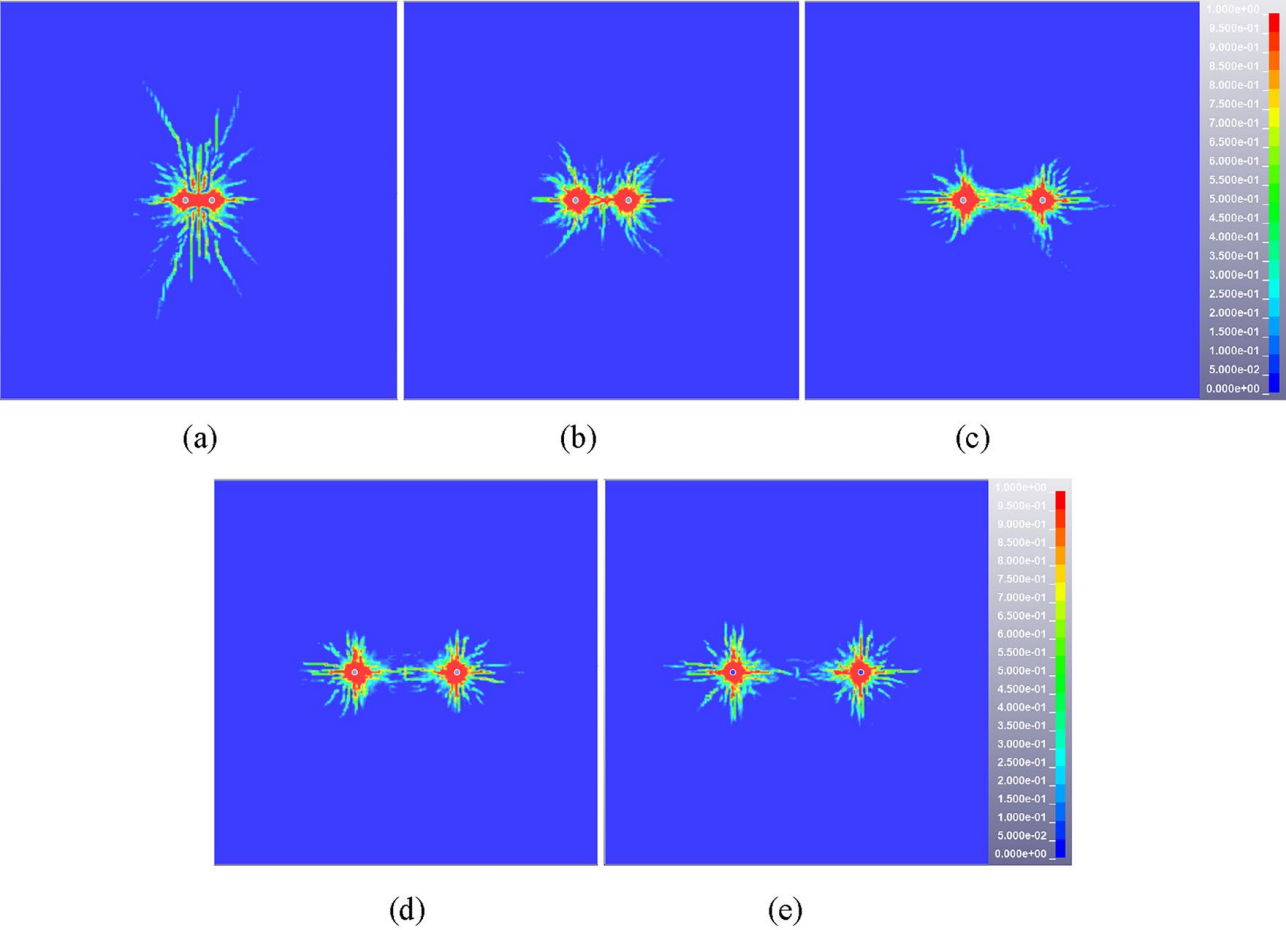
The damage distribution cloud diagram of rock mass under different blasting hole spacing (double hole blasting). (**a**) 0.2 m, (**b**) 0.4 m, (**c**) 0.6 m, (**d**) 0.8 m, (**e**) 1.0 m.

The overall damage distribution cloud diagram of rock mass in steady state is obtained by extracting the simulation results of five schemes, as seen in Fig. 9. The shape of rock damage area of each scheme is similar, showing “butterfly” distribution characteristics. However, there are still some differences in the distribution of rock damage due to the difference of borehole spacing and stress (energy) superposition area. Firstly, from the perspective of damage range and expansion direction, cracks tend to develop along the longitudinal direction and the overall damage area is band-like when the distance between the two blasting holes is relatively small, as shown in Fig. 9a. With the increase of blasting hole spacing, the transverse development of cracks gradually dominates and the longitudinal damage gradually decreases, as shown in Fig. 9b, c and d. As the spacing between the two holes increases to 1.0 m, there is no significant interaction between the two blasting holes. The damage area is basically not connected and the crack development between the holes is insufficient, as shown in Fig. 9e. Secondly, from the perspective of damage degree, the damage degree of rock mass between holes decreases with the increase of blasting hole spacing. The rock mass between the two holes changes in the form of completely broken state, full crack development state and crack non-expansion state.

**Figure 10:**
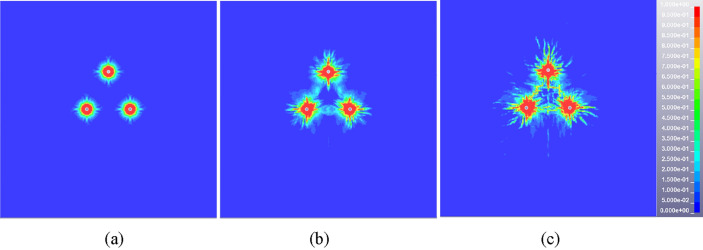
Typical time damage cloud diagram of rock mass in triple eye blasting model (hole spacing 0.6 m), (**a**) 50 μs, (b) 100 μs, (c) 500 μs.

**Figure 11:**
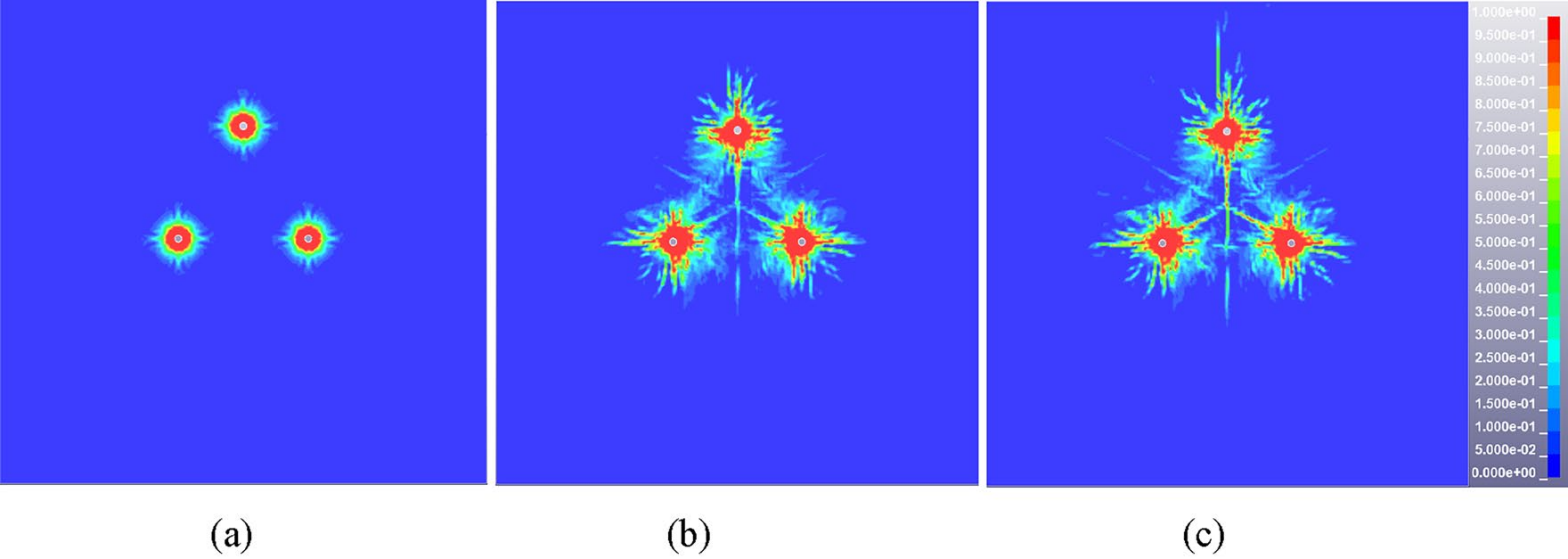
Typical time damage cloud diagram of rock mass in triple eye blasting model (hole spacing 0.8 m). (**a**) 50 μs, (**b**) 100 μs, (**c**) 500 μs.

The single row of holes cannot meet the overall blasting demand due to the longitudinal extension and thickness variation characteristics of the igneous rock intrusion area. According to the damage cloud diagram of double-hole blasting rock mass (see Fig. 9), the damage degree of the upper and lower areas of rock mass is insufficient. The triple eye blasting holes arrangement mode can not only guarantee the rock mass in this area meets the requirements of blasting pre-splitting, but also ensure the maximum utilization of explosion energy to reduce the number of blasting holes. Figures 10 and 11 show the damage evolution cloud diagram of two adjacent blasting holes with 0.6 m and 0.8 m spacing. At 100 μs after detonation, the three-hole damage area showed obvious connection. With time going by, the damage area and degree of rock mass increase continuously until it reaches a relatively stable state at about 500 μs. Both schemes can achieve good blasting results judging from the final rock mass damage results.

In conclusion, the spacing of blasting holes can significantly affect the blasting effect of igneous rock. The smaller hole spacing (0.2 m, 0.4 m) will make the rock mass between the holes completely or partially broken, resulting in a large number of broken rock mass collapse, while the larger blasting hole spacing (1.0 m) cannot guarantee good blasting effect. Therefore, the spacing of blasting holes should be greater than the superimposed stress crushing zone in practical engineering applications. The damage degree of rock mass is appropriate and the blasting effect is significant when the hole spacing ranges from 0.6 to 0.8 m. In particular, considering factors such as blasting effect and cost under different hole spacing, the hole spacing should be selected as a relatively small value of 0.6 m when it is necessary to ensure crack development and fracture degree of rock mass. When the influence range of blasting presplitting is mainly considered to reduce the number of holes, the hole spacing should be a larger value of 0.8 m. In this paper, the reasonable range of blasting hole spacing can better meet the needs of different engineering backgrounds and influencing factors for the change of hole arrangement scheme.

## Discussion

### Damage evaluation based on fractal theory

The distribution characteristics of rock burst cracks meet the fractal principle, and the fractal dimension can be used to reveal the rock blasting mechanism and reasonably assess the damage degree of rock mass. According to the crack distribution characteristics of damage cloud map, the box-counting dimension is adopted to study the damage degree of blasting rock mass^36,37^. According to the basic principle of box-counting dimension, the fractal dimension of any non-empty bounded target set can be expressed by Eq. (3)^38^.

3 $$D_{f} = \mathop {\lim }\limits_{k \to \infty } \frac{\lg N}{{ - \lg M}}$$

where *D* is the fractal dimension; *M* is a decreasing sequence with the square grid side length as the element; *N* is the minimum number of grids required to cover the target geometry with a square grid with side length.

**Figure 12:**
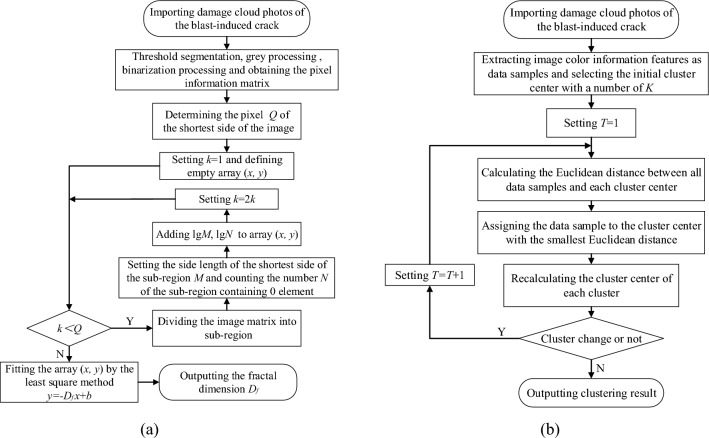
Box-counting dimension method and K-means clustering method calculation process. (**a**) Box-counting dimension method, (**b**) K-means clustering method.

Figure 12a shows the calculation process of fractal dimension of explosive crack. The fractal dimension obtained by calculation can reflect the characteristics of crack distribution and propagation. In order to quantitatively describe the damage degree of blasting rock mass, the relationship between fractal dimension and rock mass damage degree is further established, as shown in Eq. (4)^39, 40^.

4 $$\omega { = }\frac{{D_{f} - D_{0} }}{{D_{f}^{\max } - D_{0} }}$$

Where *ω* is the fractal damage; *D*_*f*_ is the fractal dimension of blast-induced cracks after blasting; *D*_0_ is the initial fractal dimension before blasting; $D_{f}^{\max }$ is the fractal dimension under complete damage and destruction, it is equal to 2 in the two-dimensional case and 3 in the three-dimensional case.

### Damage evaluation based on K-means clustering algorithm

Based on the color features corresponding to different damage values in blasting damage cloud image, K-means clustering algorithm is used to classify cloud image pixels with similar color features. The proportion of different color clusters is calculated to obtain the corresponding damage rate, so as to evaluate the feasibility and effectiveness of the blasting scheme. Damage rate refers to the proportion of the effective damage area to the total area of the plane, in which the area with damage value greater than 0.1 is regarded as the effective damage area.

K-means algorithm belongs to unsupervised learning and is a partition algorithm in cluster analysis^41,42^, the calculation process of the algorithm is shown in Fig. 12b. The core idea of K-means algorithm is to randomly select *K* initial cluster centers *C*_*i*_(1 ≤ *i* ≤ *K*) in the data set, and then use simple distance measures such as Euclidean distance to reflect the differences between the two elements. Given a sample space, the Euclidean distance calculation formula for the difference between the cluster center and the sample in space is shown as follows. By comparing the sample *X*_*i*_ with the cluster center *C*_*i*,_ the sample is classified into the cluster where the cluster center with the smallest difference is located under the condition of satisfying the Eqs. (5 and 6)^43–45^.

5 $$d\left( {X,Ci} \right) = \sqrt {\sum\limits_{j = 1}^{m} {\left( {x_{j} - C_{ij} } \right)}^{2} }$$

6 $$d\left( {Xi,Ci} \right) = \min \left\{ {D\left( {Xi,Ci} \right),i = 1,2,...n} \right\}$$

### Application example

According to the research conclusions in section "Numerical simulation results and analysis", the principle of blasting hole arrangement in igneous rock intrusion area can be preliminarily determined: the optimal distance between blasting hole and igneous rock intrusion boundary is 0.3 m; the blasting hole spacing is 0.6–0.8 m; lesser blasting holes arrangement on the premise of satisfying the blasting effect. Taking the blasting in the igneous rock intrusion area of the Zhangshuanglou Coal Mine 21,914 working face as a case study, the blasting hole layout scheme is discussed and the blasting damage effect is reasonably evaluated using box-counting dimension and K-means clustering methods.

**Figure 13:**
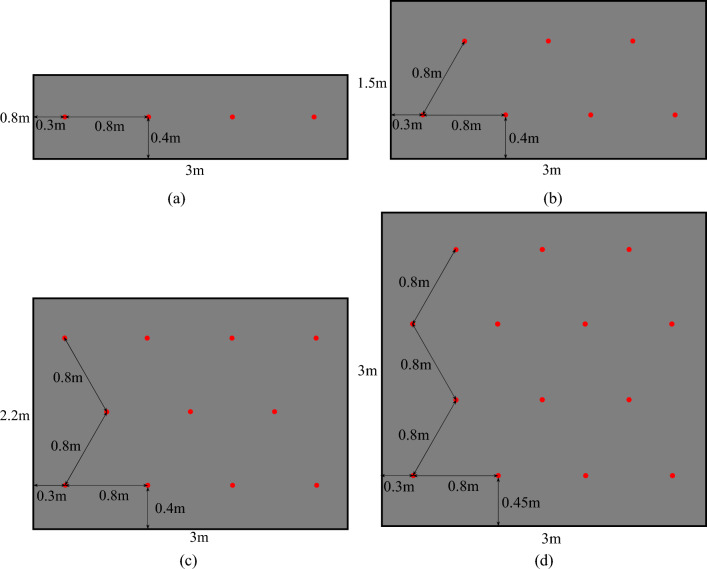
The blasting hole layout scheme under different thickness of igneous rock intrusion zone. (**a**) The thickness is 0.8 m, (**b**) the thickness is 1.5 m, (**c**) the thickness is 2.2 m, (**d**) the thickness is 3.0 m.

The 21,914 working face passes through two igneous rock intrusion areas during the mining process. One of them is located near the outer cutting hole of the working face, which will not have a significant effect on mining. The other area is located near the design stop-mining line of the working face and extends from the roof to the floor with a thickness variation of 0–3.0 m, affecting the mining range of more than 120 m. Moreover, there is a significant dividing line at the coal-rock junction to form a structural plane, which has a certain influence on the layout of blasting holes in this area. Referring to the relevant working face blasting engineering cases, the scheme design is carried out with the length of 3 m for each cycle. The blasting aperture and hole depth are 0.04 m and 1.2 m to meet the need of cutting coal twice at a time. The two rows of blasting holes near the upper and lower borders can be tilted up and down about 20° as required, and the middle row is arranged horizontally. The blasting explosive is selected as the coal mine secondary permitted water glue explosive, and the blasting is carried out by the way of forward charge and series initiation. When the thickness of the igneous rock intrusion area is less than 0.8 m, the single row blasting hole arrangement with hole spacing of 0.8 m is adopted, as shown in Fig. 13a. The double row and triple eye blasting hole arrangement with hole spacing of 0.8 m is adopted when the thickness ranges from 0.8 to 1.5 m, and the triple row and triple eye blasting hole arrangement corresponds to the thickness of 1.5 m to 2.2 m, as seen in Fig. 13b and c. The quadruple row and triple eye blasting hole arrangement scheme is adopted in the thickness range of 2.2–3.0 m, as seen in Fig. 13d. The specific blasting hole position can be adjusted according to the change of igneous rock thickness, but it is necessary to meet the above principles as far as possible.

**Figure 14:**
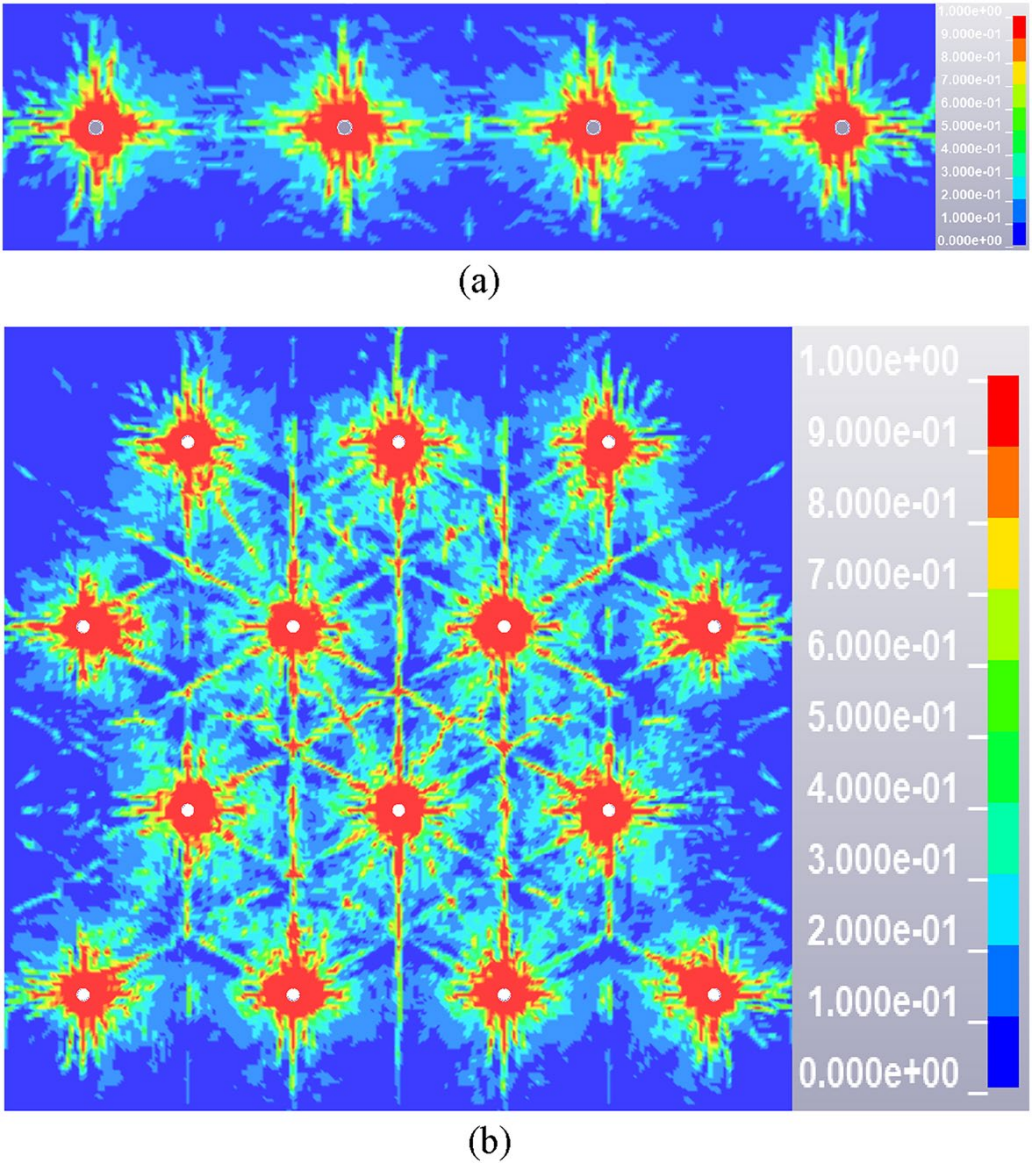
Cloud diagram of blasting damage distribution under different thickness of igneous rock intrusion zone. (**a**) The thickness of the igneous rock intrusion zone is 0.8 m, (**b**) The thickness of the igneous rock intrusion zone is 3.0 m.

According to the above igneous rock intrusion area blasting hole layout scheme, numerical simulation is carried out to study the igneous rock blasting effect under different schemes. Since the hole spacing of the four schemes is set at 0.8 m, only the results of igneous rock thickness of 0.8 m and 3 m are further analyzed, as shown in Fig. 14. In order to accurately evaluate the blasting effect of the above blasting scheme, the damage rate is calculated for quantitative analysis. Damage rate refers to the proportion of the effective damage area to the total area of the plane, in which the area with damage value greater than 0.1 is regarded as the effective damage area.

**Figure 15:**
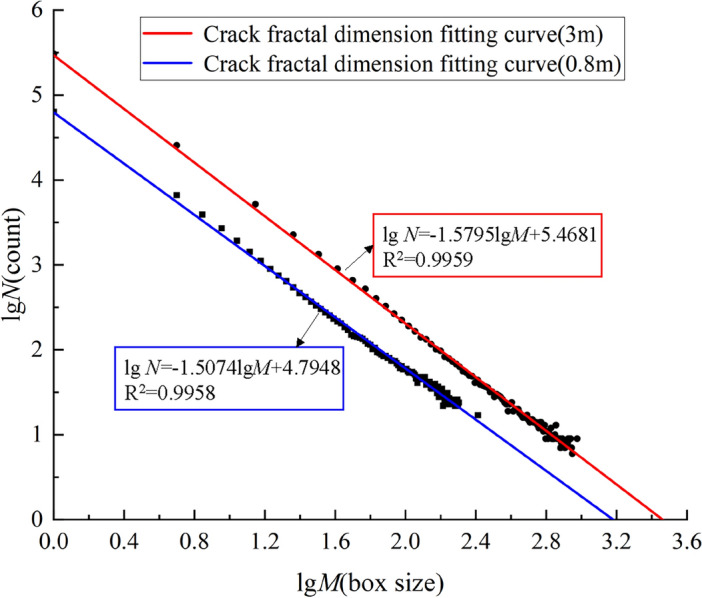
The crack fractal dimension fitting curves.

The blasting damage cloud image (Fig. 14) was filtered and binarized and imported into the calculation program of the box-counting dimension method for calculation, the crack fractal dimension fitting curves are shown in the Fig. 15. The crack box-counting dimensions of Fig. 14a and b are 1.5074 and 1.5795 respectively. The fractal damage degree of blasting is 0.7537 and 0.7898 by substituting Eq. (5). Based on the color distribution characteristics corresponding to different damage degrees in the same blasting damage cloud image, the number of color clusters is determined to be 10. Then the K-means clustering method is used to calculate the damage degree, as shown in the Table 6, the damage degree can be obtained as 0.7057 and 0.7305 respectively. Due to the defects of binarization image processing method, the box dimension method cannot distinguish the region with damage degree less than 0.1, so the result is larger than the clustering algorithm. However, the damage degree of both is above 0.7, and the deviation is within the reasonable error range, which indicating that the scheme can achieve the purpose of blasting presplitting of igneous rock and the evaluation method can quantitatively analyze the damage degree of rock mass.

**Table 6:** Classification and proportion statistics of each color cluster.

Type of cluster	Fig. 14a (thickness 0.8 m)	Fig. 14b (thickness 3 m)
(vibrant blue)	23.17% (The damage value is less than 0.1)	21.14% (The damage value is less than 0.1)
(lightish blue)	6.26% (The damage value is less than 0.1)	5.81% (The damage value is less than 0.1)
(brilliant cyan)	14.33%	14.73%
(medium aquamarine)	13.54%	13.86%
(luminous vivid malachite green)	8.82%	9.56%
(neon green)	9.36%	9.84%
(light grass green)	7.87%	7.98%
(golden yellow)	5.45%	5.45%
(ogre odor)	4.52%	4.78%
(luminous vivid red)	6.68%	6.85%

## Conclusion

1. Based on the basic mechanical parameters of igneous rocks, the mechanism of stress wave reflection and stretching effect induced by the occurrence of structural plane on the aggravation of presplitting blasting degree is determined by numerical simulation. The reflection tensile failure effect of rock mass gradually increases with the decrease of the distance between the blasting hole and structural plane.
2. The multi-hole blasting model of rock mass with different hole spacing is established to explore the superimposed stress wave transfer law of adjacent blasting holes and spatiotemporal evolution characteristics of damage. With the enhancement of the distance between blasting holes, the damage range of rock mass increases while the damage degree decreases. In addition, the damage area of rock mass under steady state presents non-uniform “butterfly” distribution characteristics.
3. The principle of blasting hole arrangement in igneous rock intrusion area is determined by relevant numerical simulation analysis results: the optimal distance between blasting hole and igneous rock intrusion boundary (or structural plane) is 0.3 m; the blasting hole spacing is 0.6–0.8 m; lesser blasting holes arrangement on the premise of satisfying the blasting effect. The blasting scheme is designed based on the igneous rock intrusion area of 21,914 working face.
4. The box-counting dimension and K-means clustering methods are used to reasonably evaluate the effect of rock mass presplitting blasting. The results show that the effective damage degree of rock mass obtained by the two methods is both greater than 0.7 and the evaluation methods are reliable. The design scheme can achieve the purpose of presplitting blasting in igneous rock area and guarantee the normal mining of the working face.

## Data Availability

The authors declare that the data supporting the findings of this study are available within the paper. Should any raw data files be needed in another format they are available from the corresponding author upon reasonable request.
